# Is Cortical Theta-Gamma Phase-Amplitude Coupling Memory-Specific?

**DOI:** 10.3390/brainsci12091131

**Published:** 2022-08-25

**Authors:** Orestis Papaioannou, Laura P. Crespo, Kailey Clark, Nicole N. Ogbuagu, Luz Maria Alliende, Steven M. Silverstein, Molly A. Erickson

**Affiliations:** 1 Department of Psychiatry & Behavioral Neuroscience, University of Chicago, Chicago, IL 60637, USA; 2 Departments of Psychiatry, Neuroscience and Ophthalmology, University of Rochester, Rochester, NY 14627, USA

**Keywords:** theta-gamma phase-amplitude coupling, working memory, EEG, phase-amplitude coupling

## Abstract

One of the proposed neural mechanisms involved in working memory is coupling between the theta phase and gamma amplitude. For example, evidence from intracranial recordings shows that coupling between hippocampal theta and cortical gamma oscillations increases selectively during working memory tasks. Theta-gamma phase-amplitude coupling can also be measured non-invasively through scalp EEG; however, EEG can only assess coupling within cortical areas, and it is not yet clear if this cortical-only coupling is truly memory-specific, or a more general phenomenon. We tested this directly by measuring cortical coupling during three different conditions: a working memory task, an attention task, and a passive perception condition. We find similar levels of theta-gamma coupling in all three conditions, suggesting that cortical theta-gamma phase-amplitude coupling is not a memory-specific signal, but instead reflects some other attentional or perceptual processes. Implications for understanding the brain dynamics of visual working memory are discussed.

## 1. Introduction

Working memory, or the short-term storage of information in service of a goal, is one of the core processes in cognition; it is responsible for representing and actively maintaining information in a flexible manner and making it available to other central cognitive processes [1,2]. As such, working memory is associated with multiple indicators of overall cognitive capacity within the general population, including academic success [3] and IQ [4,5]. Conversely, a low working memory capacity has been linked to poorer outcomes in people with serious mental illnesses such as schizophrenia [6,7]. However, despite the significance of working memory for both understanding general cognition and predicting mental health outcomes, the specific brain dynamics involved have proven elusive and remain poorly understood.

**Figure 1:**
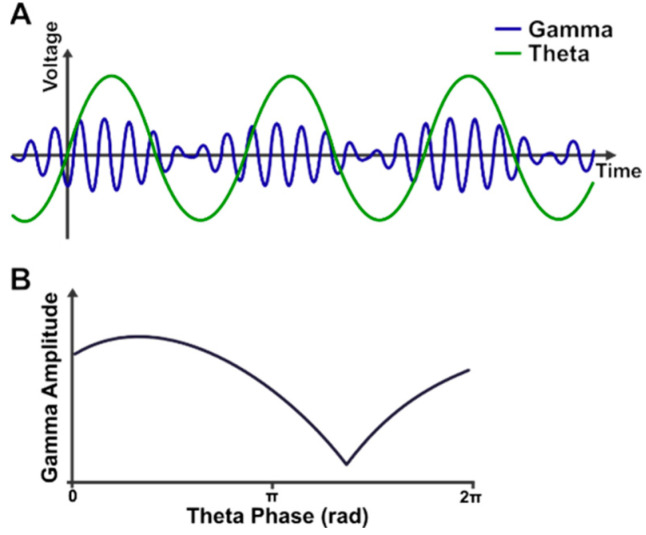
Schematic illustration of theta-gamma phase-amplitude coupling (**A**), with the gamma amplitude being modulated by the theta phase. (**B**) shows the average gamma amplitude (i.e., the average envelope amplitude) at different theta phases. Note that the peak gam.

One putative mechanism of working memory that has garnered interest in recent years is theta-gamma phase-amplitude coupling—that is, the amplitude of gamma (30–100 Hz) oscillations is modulated by the phase of lower-frequency theta (4–8 Hz) oscillations (see Figure 1 for a schematic representation of this effect). Under this model, information is represented in local network gamma oscillations, and these local networks are coordinated via long-distance theta oscillations. This allows multiple units of information to be maintained concurrently by containing each unit within a particular part of the theta cycle [8,9,10].

The strongest evidence for theta-gamma phase-amplitude coupling as a mechanism for working memory comes from intracranial recordings. Evidence from both iEEG [8,11] and electrocorticography (ECoG) [12] shows that theta-gamma phase-amplitude coupling—especially coupling between the hippocampal theta and cortical gamma—is elevated during a visual working memory task. Specifically, the degree of hippocampal-to-prefrontal cortex theta-gamma coupling is modulated by the demands on working memory, with higher coupling being observed when participants were asked to remember more than one item [13]. This suggests that theta-gamma coupling between subcortical and cortical areas plays a crucial role in working memory maintenance, consistent with theoretical conceptualizations of theta serving as a carrier for information-dense gamma activity [9,10].

Although intracranial recordings provide strong evidence for the functional role of theta-gamma phase-amplitude coupling, the invasive nature of the technique means that the available data are limited to certain patient populations or animal studies, and sample sizes are often quite small. As such, interest has grown in the measurement of theta-gamma phase-amplitude coupling through the use of noninvasive techniques such as electroencephalography (EEG) e.g., [14] or magnetoencephalography (MEG) e.g., [15]. Importantly, this is conceptually different from the hippocampus-driven theta-gamma phase-amplitude coupling observed in iEEG and ECoG, as scalp EEG can only measure activity originating in cortical structures, and MEG has a much lower sensitivity for activity originating from subcortical structures. Furthermore, the overall signal-to-noise ratio for gamma activity is much lower in EEG recordings than in intracranial recordings [16], adding further complications to the task of measuring theta-gamma phase-amplitude coupling noninvasively.

Nevertheless, cortical (EEG/MEG) theta-gamma phase-amplitude coupling has shown some promise as a marker of working memory. For example, multiple studies have shown elevated theta-gamma phase-amplitude coupling during the delay period or response window for various working memory tasks (e.g., [14,17,18]); though, unlike in studies using iEEG and ECoG, cortical phase-amplitude coupling does not seem to be sensitive to the working memory load in these studies. Furthermore, patients with Alzheimer’s disease [19] or schizophrenia [20] who exhibit impaired working memory show less theta-gamma phase-amplitude coupling overall than healthy control participants at rest, with the degree of theta-gamma phase-amplitude coupling being related to measures of cognitive impairment at the group level [19,20]. Taken together, these studies suggest that cortical theta-gamma phase-amplitude coupling can indeed be measured in the scalp during working memory tasks, and disruption of these fundamental brain dynamics may be important for understanding cognitive impairment and patient outcomes. 

Despite the above observations, the specificity of the relationship between coupling and working memory has not yet been established. For example, the available studies only include comparisons between the baseline (where no stimulus is present) and the delay period (after the presentation of items to be stored in memory); therefore, it is unclear whether theta-gamma synchronization reflects a memory-specific process, as opposed to sensory or perceptual processes, attentional allocation, and/or other factors. In fact, it has been suggested that at least some of the observed theta-gamma coupling reflects sensory-related phase resetting that occurs following the onset of a visual stimulus (see [21] for a discussion). Furthermore, unlike hippocampus-based coupling, there has not been a clear association between cortical theta-gamma coupling and behavioral performance [17,21]. 

In this study we investigated the degree to which cortical theta-gamma phase-amplitude coupling is specific to working memory by measuring coupling during three tasks: a change-detection task (that requires working memory maintenance), a visual attention task (that requires central processes but not the maintenance of information), and a passive viewing task (where the stimuli were completely task-irrelevant). Crucially, the stimulus sequence in each of the three tasks was nearly identical, permitting the dissociation of activity related directly to working memory from that related to other non-memory-related processes. If cortical theta-gamma coupling is truly memory-specific, then we would expect that the working memory task would yield significantly elevated coupling compared to the other two tasks. Conversely, if theta-gamma coupling is related to some more general process—e.g., if it is a result of spatial attention [15] or perhaps sensory processing [22]—then we would expect to see similar levels of coupling across all three tasks. 

## 2. Methods

This study constitutes a reanalysis of data previously reported by Erickson et al. 2019 [23] as well as a new second sample that allowed us to test the replicability of our effects. A full description of the paradigm, stimuli, and methods can be found in the original publication, although we will describe the task briefly here. 

### 2.1. Participants

A total of 66 participants were recruited from Rutgers University and the surrounding community. Following artifact rejection, 6 participants were rejected from the analysis, so the final sample consisted of 60 participants (36 female). All participants were between the ages of 18 and 60 (M = 35.85, SD = 13.58) and self-reported no history of mental illness. They also reported that they had no first-degree relatives with a psychotic disorder, had normal or corrected-to-normal vision, and were free from a history of developmental and neurological disorders. All participants had normal or corrected-to-normal vision.

Additionally, a second sample of 20 participants (11 female) was recruited from the University of Chicago to examine the replicability of the observed effects from the primary sample. All participants in this replication sample were between the ages of 18 and 65 (M = 36.82, SD = 9.31) with no history of mental illness, neurological disorders, or developmental disorders. This participant group also had no first-degree relatives with a psychotic disorder and exhibited normal or corrected-to-normal vision. Participants from both samples were reimbursed for their time, and informed consent was obtained prior to their participation in the experiment. All recruiting and experiment procedures were approved by the Rutgers University Institutional Review Board and the University of Chicago Institutional Review Board, respectively. 

### 2.2. Experimental Paradigm

**Figure 2:**
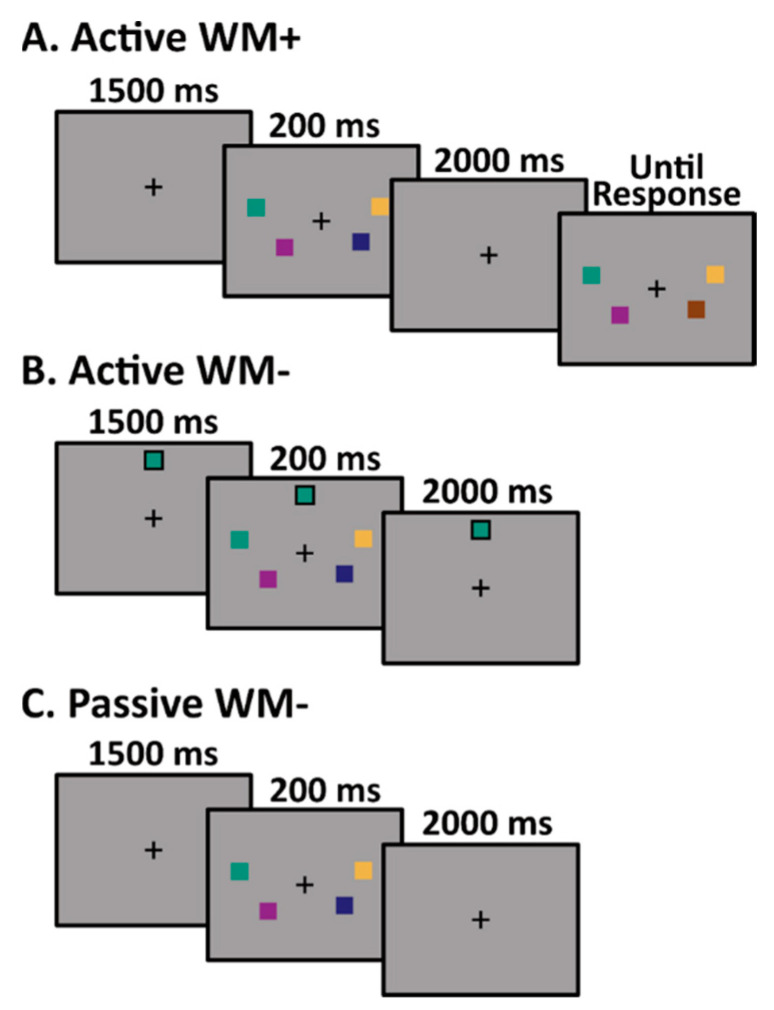
Trial sequence for the three tasks: (**A**) Active WM+, (**B**) Active WM-, and (**C**) Passive WM-.

Each participant completed three task variants using similar stimulus sequences. The first task (Active WM+; Figure 2A) was a change-detection task designed to assess visual working memory capacity [24,25]. On each trial, a sample array consisting of two, four, or six colored squares was presented around a central fixation cross for 200 ms. The sample array was followed by a 2000 ms delay period during which participants were asked to retain the colors of the squares in memory. Finally, a test array was presented until the response. On 50% of the trials, all of the colors in the test array were identical to the colors from the sample array. On the other 50% of the trials, one of the squares switched to a new color. Participants were asked to indicate by a button-press response whether or not the test array matched the sample array. 

The second task (Active WM-; Figure 2B) consisted of a nearly identical stimulus sequence. In this task, participants were required to actively attend to the stimuli from the 200 ms sample array in search of a target color, but were not required to store any of the colors in working memory. In order to eliminate all memory requirements from this task, a single square depicting the target color was continuously present at the top of the screen. When a sample array contained a square matching the target color (10% of trials), participants were instructed to press a button on a gamepad. No response was required on the 90% of trials in which a target color was absent from the sample array. 

The third task (Passive WM-; Figure 2C) again consisted of a nearly identical stimulus sequence to the Active WM+ and Active WM- task variants. In this task, participants were instructed to passively view the sample arrays. An unrelated simple intermittent task was used to ensure adequate engagement. 

This same experimental design was used for the replication sample, with the exception that sample arrays consisted of 1, 2, 3, 4, or 5 colored squares, rather than 2, 4, or 6 colored squares. Furthermore, the test arrays in the Active WM+ condition only included one of the squares (with the participant reporting whether that particular square was the changed color or not). 

### 2.3. EEG Recording and Pre-Processing

For the primary sample, EEG data were recorded continuously at 1000 Hz using a Brain Products ActiCHamp system (Brain Products GmbH, Gilching, Germany) over 64 channels (60 scalp electrodes, 3 EOG electrodes, and 1 online reference electrode). Data were referenced online to a single electrode on the tip of the nose, and an online anti-alias low-pass filter at 200 Hz was applied. The replication sample was also recorded continuously at 1000 Hz using a Brain Products ActiCHamp system over 64 channels. For this sample, data were referenced online to a single electrode (P9, similar to the left mastoid), and an online anti-aliasing low-pass filter at 200 Hz was again applied.

Offline processing was performed in Matlab (Mathworks Inc., Natick, MA, USA) using the EEGLAB [26] and ERPLAB [27] toolboxes. Data from the primary sample were first re-referenced offline to the average of the left and right mastoids, whereas the replication sample data were re-referenced to the average of P9 and P10, which is a close approximation to the left and right mastoids. Data were then segmented to −1500–2500 ms relative to the onset of the stimulus array, and adjusted with respect to the pre-stimulus baseline. Abnormally noisy channels were identified by visual inspection and interpolated. ICA correction was performed to remove components related to eye movements and eye blinks, and artifact correction was performed to remove trials with abnormally large voltage deflections (>150 μV peak to peak). No offline filtering was applied at this stage. 

### 2.4. Theta-Gamma Phase-Amplitude Coupling

Theta-gamma phase-amplitude coupling was then performed on the preprocessed data using the procedure described in Sauseng et al. [14]. Briefly, the EEG segments were first converted from voltage to current-source density (CSD) using an ERPLAB inbuilt function. Theta-gamma phase-amplitude coupling was measured during two time windows: (1) during the pre-stimulus (baseline) period of −940 to −200 ms, prior to stimulus onset; and (2) during the post-stimulus period (300 to 1900 ms after stimulus onset). The following procedures were then conducted separately for each of the two time windows: first, the FieldTrip Matlab toolbox [28] was used to calculate the instantaneous amplitude and phase at 2–70 Hz. Specifically, we used a 5-cycle Morlet waveform transformation, resulting in a complex decomposition of the data, with the amplitude and phase angle depicted by the real and imaginary part of the transformed complex value, respectively. For the present analysis, we measured the theta phase at 6 Hz, and the gamma amplitude in a window of 20–70 Hz at 1 Hz intervals. Note that this range includes frequencies that are lower than the traditional gamma range (namely 20–30 Hz). These frequencies were included in the analysis to more closely match the methods described by Sauseng et al. 2009. As discussed further in the Results, two gamma ranges of interest emerged (25–35 Hz and 45–55 Hz), hereafter referred to as ‘low’ and ‘high’ gamma, respectively. These two bands were thus used in our statistical analyses. 

**Figure 3:**
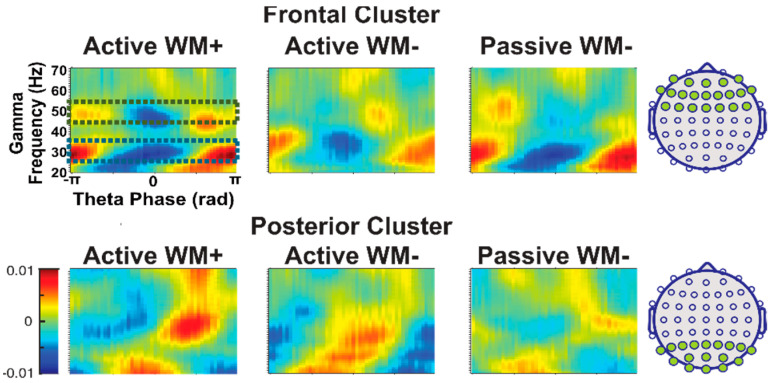
Average normalized gamma amplitude at each theta phase across our two clusters of interest, averaged across all 60 subjects of the primary sample.

To visually represent the coupling, we first normalized the gamma amplitude in each trial, then binned the gamma amplitude by the concurrent theta phase (separately for each electrode, condition, and time window), and plotted the average gamma amplitude at each theta phase. For ease of visualization, a frontal cluster and posterior cluster were created by averaging together the binned data of 21 frontal channels and 17 posterior and occipital channels, separately. See Figure 3 for a representation of all the channels included in each cluster. 

In order to numerically compare coupling across the different conditions and time windows, we calculate the Phase Locking Value (PLV) as described in Sauseng et al. 2009 [14] and Penny et al. 2008 [29]. We first calculated the amplitude envelope of each frequency in our gamma range (20–70 Hz) and then used a Hilbert transform to extract the phase information of the gamma amplitude fluctuations. Finally, PLV was then calculated using the following formula:

(1) $$PLV=\left|\frac{1}{N}\overset{N}{\underset{n=1}{\sum }}\exp\left(i\left(\varphi_{\vartheta }\left[n\right]-\varphi_{A_{\gamma }}\left[n\right]\right)\right)\right|$$

where $\varphi_{\vartheta }\left[n\right]$ is the theta phase angle at a given time point, and $\varphi_{A_{\gamma }}\left[n\right]$ is the phase angle of the gamma amplitude envelope. This was used to estimate the coherence between the phase of the gamma amplitude envelope and the theta phase for each condition, electrode, and trial independently. PLV was then averaged across trials to obtain a single PLV estimate per condition and electrode. Frontal and posterior clusters were then created by averaging PLV across the corresponding electrodes. Note that since the pre-stimulus time window included fewer timepoints than the post-stimulus period window, we used a random permutation approach to splice together time points from the pre-stimulus window to achieve a similar number of total time points per segment for the post-stimulus window. Lastly, to test the possibility that the observed PLV was obtained by chance, this analysis was repeated with shuffled theta phase data, creating a null comparison condition.

### 2.5. Statistical Analyses

Statistical analyses were performed using the R-studio development suite [30] and R statistical software [31], including specific packages for conducting repeated measures of ANOVA [32], calculating Bayes factors [33], and conducting linear mixed effect models [34,35] for the main results.

Our main statistical analyses rely on the following planned comparisons: first, we conducted four 2 × 3 (time window × task) repeated measure ANOVAs to determine whether PLV changed significantly between the pre-stimulus and post-stimulus delay periods. This was done separately for the frontal and posterior clusters and for the low and high gamma band. These same four comparisons were also conducted for the replication sample. Where applicable, Greenhouse–Geisser corrections for sphericity violations were applied; the degrees of freedom reported below thus reflect these statistical corrections.

Second, a within-subjects Bayes Factor analysis was performed to examine the strength of evidence for the main effect of the task and time window separately for the two clusters, frequency bands, and samples. This allowed us to discern whether there is strong evidence for a null effect (or main effect) or simply a lack of evidence for the presence of an effect.

Third, a linear mixed effect model was used for a more focused contrast analyses to test the specific hypothesis that PLV in the Active WM+ condition was higher than for the other two conditions overall. This focused approach allows for greater power for detecting the specified contrast, but does not test for any other effects of conditions (e.g., a difference between Active WM- and Passive WM-).

Lastly, Pearson correlations were calculated to assess the covariation of individual capacity scores (K) and the average PLV during the delay period in the Active WM+ condition, separately for the primary and replication sample. Due to the different test arrays used in the original experiment and the replication experiment, we used slightly different formulas to estimate capacity in each experiment [36]. The main experiment used a full display in the test array, so we used the more appropriate Pashler’s formula to estimate K, where $K=S\left(\frac{h-f}{1-f}\right)$, with S being the set size of the sample array, and h and f being the hit rate and false alarm rate for that set size, respectively. The replication experiment used a single probe test array. Thus, for the replication sample we used Cowan’s K formula, where $K=S\left(h-f\right)$, as that is more appropriate for single probe displays.

A series of exploratory pairwise comparisons were also performed to investigate any differences in the mean PLV across the two electrode clusters or across frequency bands. This was completed as a series of pairwise t-tests comparing the average PLV difference between the frontal and posterior clusters or from a low gamma to high gamma, separately for each condition. Due to the large number of comparisons and the exploratory nature of this analysis, a false discovery rate correction [37] was applied to reduce the number of false positives.

## 3. Results

### 3.1. Effects of Task and Time Window

Theta-gamma phase-amplitude coupling in frontal and posterior clusters is depicted in Figure 3. Using a collapsed localizer approach [38], we plotted the average normalized gamma amplitude at each theta phase, collapsing across all three tasks. Using visual inspection, we identified two frequency ranges of interest that showed the highest modulation by phase irrespective of condition: a low gamma band of interest (25–35 Hz) and a high gamma band of interest (45–55 Hz). Given the exploratory nature of this study, we focused our analyses on these two bands to increase our power for detecting any effects. For all analyses, we calculated the mean PLV separately for a low and high gamma.

**Figure 4:**
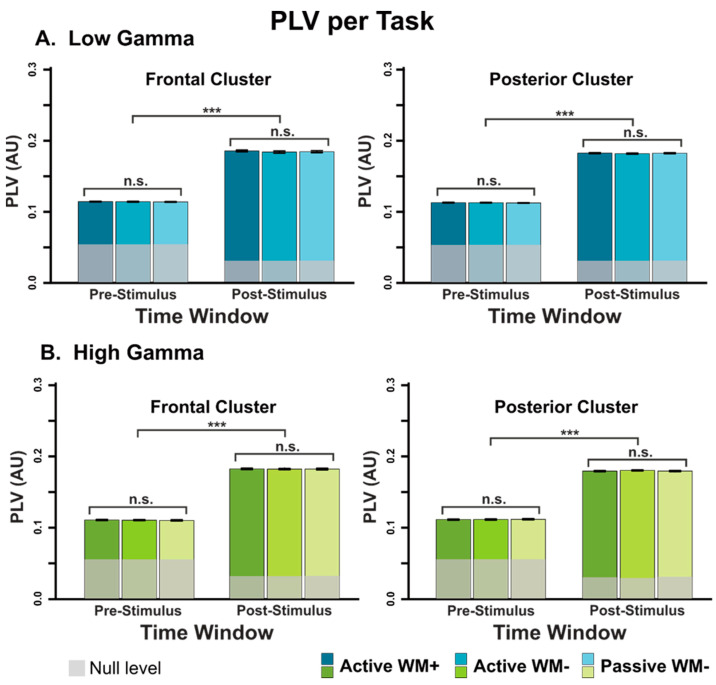
Average PLV per task across the different clusters and frequency bands. The shaded areas on each bar represent the PLV of the null permutations and mark the PLV one would expect by chance. When marking significance, *** denotes *p* < 0.001.

The results of the ANOVAS can be found in Table 1. Overall, we observed elevated PLV during the post-stimulus period compared to the pre-stimulus period irrespective of task, cluster, or frequency band (see Figure 4). Specifically, we found that the PLV was significantly higher in the post-stimulus time-window compared to the pre-stimulus time-window in both the frontal and posterior clusters and both frequency bands (*p* < 0.001, η^2^ > 0.98 in all cases). Interestingly, PLV did not significantly vary across tasks for either high or low gamma, or for frontal or posterior channels (*p*’s > 0.05, η^2′^s < 0.01), nor was there an interaction between task and time window (*p*’s > 0.17, η^2′^s < 0.01), suggesting that the cortical PLV magnitude is not impacted by task demands. To further interrogate these effects, follow-up Bayes Factor analyses found very strong evidence for the null when looking at the main effect of a task (BF_10_ < 0.034); conversely, we observed extremely strong evidence for the main effect of the time window (BF_10_ < 10^355^ in all cases). Thus, it seems that all three tasks led to increases in theta-gamma phase-amplitude coupling following the onset of the stimuli to a similar degree.

Although we generally found no evidence for the effect of tasks, one comparison (for the low gamma band in the frontal cluster) did approach significance (F(1.74,102) = 3.12, *p* = 0.055). To fully explore the dataset, we performed a series of exploratory post-hoc comparisons to examine the pattern of PLV magnitude by task. Qualitatively, we observed that post-stimulus PLV in the Active WM+ task was slightly higher than the post-stimulus PLV in the Active WM- condition (t(59) = 2.34, *p* = 0.023), with the PLV in Passive WM- condition lying between the other two conditions. However, these differences were not significant after correcting for a false discovery rate; furthermore, no such significant effects were observed in the replication sample (see below).

An identical pattern of results was found in the replication sample (see Supplementary Materials), providing further evidence that tasks did not significantly affect coupling. That is, we observed a significant main effect of the time window (*p* < 0.001, η^2^ > 0.98) but no significant effect of tasks (*p*’s > 0.09, η^2′^s < 0.01), nor a significant interaction between the time window and task (*p*’s > 0.61 η^2′^s < 0.01). Bayesian analyses provide further evidence for the pattern, with extremely strong evidence for a main effect of the time window (BF_10_ > 10^^115^ for all cases), but very strong evidence for the null when examining the main effect of tasks (BF_10_ < 0.081 in all cases).

### 3.2. Focused Contrast Analysis

Because the omnibus ANOVAs tested all pairwise comparisons and not the critical contrasts of interest, it is possible that they are not sufficiently sensitive to provide a strong test of the hypothesis that theta-gamma coupling is elevated in the active WM+ condition. We therefore additionally conducted analyses examining a more focused contrast using a linear mixed effects model—this allowed us to test the narrower hypothesis that the PLV during the active WM+ condition was specifically higher than the other two conditions, with greater power than an omnibus test. We used a linear mixed effect model with fixed effects of the time window (pre-stimulus vs. post-stimulus) and condition (Active WM+, Active WM-, and Passive WM-) and a random effect for the subject (to account for the repeated measures design). We manually specified the contrasts of the condition effect as 2/3, −1/3, and −1/3, respectively, meaning that rather than a general effect of the condition, our model was testing for a specific effect where the Active WM+ condition led to a higher PLV value than the other two conditions overall. We applied this model across the two frequency bands and electrode clusters independently (see Table 2). Similar to the ANOVA results, we found a significant effect of the time window (t(295) > 210, *p* < 0.001, Cohen d > 10^4^ in all cases) but no significant effect of the condition (t(295) < 0.86, *p* > 0.390, Cohen d < 0.04 in all cases) nor a significant interaction between the time window and condition (t(295) < 1.37, *p* > 0.171, Cohen d < 0. 11 in all cases). An identical pattern of results was found in the replication sample (see Supplemental Materials).

### 3.3. Effects of Cluster and Frequency Bands

Given the apparent differences in coupling strength between the frontal and posterior electrode clusters (see Figure 3), we conducted an exploratory series of t-tests to determine whether coupling differed by cluster location for any of the comparisons. Surprisingly, we found that coupling strength was not significantly different between posterior and frontal regions for any of the comparisons examined (see Table 3, all *p*’s > 0.060, all Cohen’s d’s < 0.25). Further inspection of the data revealed the source of these apparently contradictory observations (see Supplementary Figure S1): compared to posterior electrodes, frontal electrodes exhibited a much more consistent relationship between theta phase and peak gamma amplitude, meaning that phase-amplitude coupling is more uniform within the frontal cluster. By contrast, in the posterior cluster the specific phase at which gamma amplitude is highest differs even among neighboring electrodes—in other words, posterior coupling seems to be rather localized, while frontal coupling is more distributed. However, since the PLV calculation merely captures covariance between theta phase and gamma amplitude and is not impacted by the specific theta phase at which the gamma amplitude is highest, PLV values are similar across frontal and posterior clusters, despite the apparent differences shown in Figure 3, which depict the average gamma amplitude by theta phase across all electrodes within the frontal and posterior clusters.

We also conducted a similar set of pairwise t-tests to assess the degree to which PLV differed across the two frequency bands of interest. We found that the low gamma band yielded significantly higher PLV compared to the high gamma band in all comparisons (see Table 4, all *p*’s < 0.001, all Cohen’s d’s > 0.5). Thus, it appears that theta-gamma coupling is more robust within the lower gamma frequency band, an effect that appears equally strong for both the pre-stimulus and post-stimulus time windows. An identical pattern of results was observed in the replication sample (Supplementary Materials). Briefly, we found that PLV was statistically similar between frontal and posterior clusters for all tasks and time windows, while the low gamma band led to significantly higher PLV values than the high band across all comparisons.

### 3.4. Relationship between PLV and Working Memory Capacity

Finally, we calculated the average PLV separately for the two electrode clusters and two frequency bands during the delay period (i.e., for the post-stimulus window in the Active WM+ task), and examined the correlation between the observed individual coupling levels and Cowan’s K, a measure of individual working memory capacity in change-detection tasks [36]. A false discovery rate correction was applied to correct for multiple comparisons [37]. If theta-gamma coupling is related to working memory capacity, we would expect that participants with a higher capacity will demonstrate a stronger coupling, and thus overall larger PLV values during the delay period of Active WM+.

**Figure 5:**
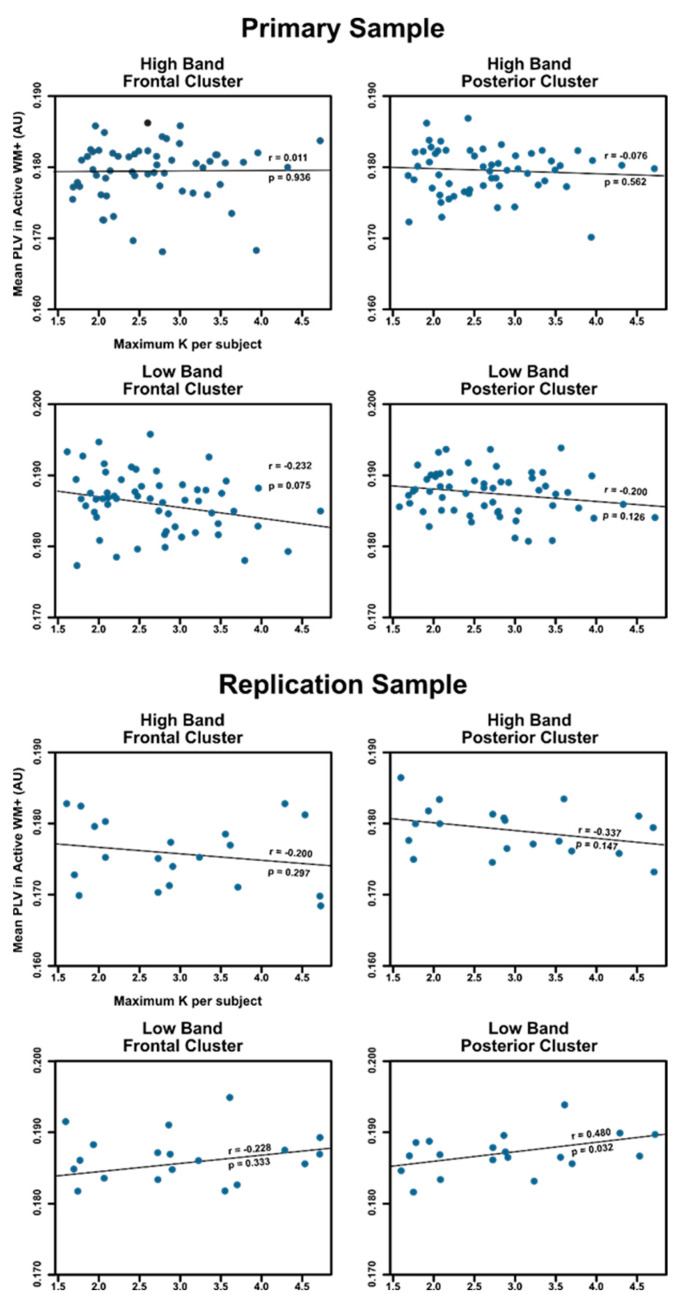
A scatterplot of individual delay period PLV and K, a measure of working memory capacity for the main sample (**left**), and the replication sample (**right**). The correlation for and *p*-value for each sample are noted on the graph, as well as the line of best fit.

We found that there was no significant association between individual PLV values and K for either the frontal or posterior electrode cluster, or for high or low gamma in either the primary sample (abs(r’s) < 0.232; *p*’s > 0.075) or the replication sample (abs(r’s) < 0.480; *p*’s > 0.032) after correction. In the replication sample, the correlation between the low band posterior cluster PLV and K was significant before correction (r = 0.480, *p* = 0.032). However, this comparison failed to remain significant after FDR correction was applied. Furthermore, the equivalent comparison in the primary sample was not significant (r = −0.200, *p* = 0.126), suggesting that the observed correlation is unlikely to reflect a consistent relationship between PLV and K. To visualize the associations between PLV and K, we plot the average PLV and K across the different clusters, frequency bands, and samples in Figure 5.

## 4. Discussion

In this study, we investigated the question of whether cortical theta-gamma phase-amplitude coupling is specific to working memory or if it reflects a broader set of processes applicable to many cognitive domains, including perception and attention. We found strong evidence that, although coupling was significantly elevated during the post-stimulus period compared to the pre-stimulus period and compared to the null model, it was elevated to the same degree across all three tasks—two of which did not require any maintenance of information in working memory. Furthermore, coupling from the memory condition was not significantly or consistently correlated with working memory capacity. This suggests that theta-gamma phase-amplitude coupling, as measured using EEG, likely reflects more widely distributed perceptual processes or obligatory phase resetting following the onset of a visual stimulus, rather than a specific cognitive process such as working memory. These conclusions are further supported by the observation that an identical pattern of results was subsequently observed in a smaller replication sample.

The findings from this study are consistent with observations from the literature that there is little association between cortical coupling and behavioral measures of working memory [17,21], and may provide an explanatory account for this lack of correlation. If cortical theta-gamma coupling reflects other processes, then it might be expected that the coupling captured by those paradigms was not necessarily reflecting the number of items stored in memory, and thus did not significantly vary with performance. Although the present observations may explain this lack of correlation, the functional significance of this signal remains unclear. Given that theta-gamma coupling is significantly elevated during the post-stimulus period compared to the pre-stimulus period, we speculate that it may serve a more generalized perceptual function. Further research is needed to better understand the conditions under which coupling can be modulated, as well as to understand the significance of reduced coupling observed in neurological and psychological disorders (e.g., [19,20]).

Although these findings may appear at odds with the original work linking theta-gamma coupling to working memory [14], there are several caveats. First and foremost, it may be that the hippocampus-to-cortex theta-gamma coupling measured through iEEG and ECoG [8,11,12] does not meaningfully translate to scalp measures like EEG. Of note, it remains possible that MEG will be able to capture memory-specific coupling whereas EEG cannot, although more research is needed to determine to what degree that holds true.

Second, theta-gamma coupling was measured within-electrode, as described by Sauseng et al. [14]. Therefore, the putative function of theta-gamma coupling (i.e., to coordinate signals across distant brain structures) might not have been captured by this analytical approach. Similarly, it could be that working memory maintenance affects the *distribution* of coupling across cortical networks but not the overall *degree* of coupling (which is our main outcome in this analysis). Given that the present analytical approach is rather insensitive to topographical differences in coupling, more advanced computational techniques such as functional connectivity analyses or multivariate decoding techniques may be used to test this hypothesis.

Finally, the working memory task used in this study relies heavily on the maintenance of perceptual information and less on the executive function; that is, a change-detection task merely requires participants to store the colors in memory but without any active manipulation or updating of the items that are characteristic of other types of working memory tasks. It is possible that other paradigms that require more executive control, such as the N-back task, may depend on theta-gamma coupling to a greater degree than simple maintenance tasks.

## 5. Conclusions

In conclusion, the current study shows strong evidence that cortical theta-gamma phase-amplitude coupling occurs to an equivalent degree in tasks that do not require working memory maintenance as it does in a task that does require maintenance. Thus, it is likely that within-electrode coupling reflects other processes, such as perceptual processing or spatial attentional control, and not working memory per se. Although it is possible that a memory-specific signal does exist in the dynamics of theta or gamma oscillations, further research is needed to isolate such a signal from more general neural dynamics. Similarly, more research is required to identify which processes modulate cortical theta-gamma phase-amplitude coupling, and to what degree hippocampal theta interacts with cortical coupling.

## Figures and Tables

**Table 1:** Results from the task × time window ANOVAs across the different electrode clusters and frequency bands. Note that for the main effect of the task and the task-by-time-window interaction, we report the corrected degrees of freedom and *p*-values. No sphericity correction was necessary for the main effect of the time window since it only has two levels. When marking significance, *** denotes *p* < 0.001.

Main ANOVA Results							
Cluster	Frequency Band	Effect	dfs	F	*p*(GG)	η^2^	BF10
Frontal	High	Time Window	1, 59	73,073	<0.001 ***	0.986	>10^374^
Frontal	High	Task	1.97, 116.3	0.40	0.666	0.006	0.030
Frontal	High	Timewindow × Task	1.96, 115.4	0.27	0.757	0.002	--
Frontal	Low	Time Window	1, 59	20,363	<0.001 ***	0.995	>10^355^
Frontal	Low	Task	1.74, 102.8	3.12	0.055	0.044	0.031
Frontal	Low	Timewindow × Task	2.00, 118.0	2.57	0.081	0.003	--
Posterior	High	Time Window	1, 59	5915	<0.001 ***	0.980	>10^374^
Posterior	High	Task	1.97, 116.1	0.18	0.831	0.008	0.033
Posterior	High	Timewindow × Task	1.88, 111.1	1.77	0.177	0.000	--
Posterior	Low	Time Window	1, 59	5625	<0.001 ***	0.990	>10^403^
Posterior	Low	Task	1.91, 112.7	0.79	0.451	0.001	0.030
Posterior	Low	Timewindow × Task	1.91, 112.6	1.54	0.217	0.004	--

**Table 2:** Results from the task × time window linear mixed model analysis using the focused contrasts across the different electrode clusters and frequency bands. Note that for the effect of tasks, we only tested the narrow hypothesis that Active WM+ led to a higher PLV than the other two conditions. When marking significance, *** denotes *p* < 0.001.

Contrast Analysis Results						
Cluster	Frequency Band	Effect	df	t	*p*	Cohen’s d
Frontal	High	Time Window	295	240	<0.001 ***	>10^4^
Frontal	High	Task	295	0.86	0.390	0.043
Frontal	High	Timewindow × Task	295	−0.28	0.782	0.005
Frontal	Low	Time Window	295	210	<0.001 ***	>10^4^
Frontal	Low	Task	295	0.66	0.512	0.025
Frontal	Low	Timewindow × Task	295	1.37	0.171	0.109
Posterior	High	Time Window	295	240	<0.001 ***	>10^4^
Posterior	High	Task	295	−0.85	0.395	0.042
Posterior	High	Timewindow × Task	295	0.68	0.495	0.027
Posterior	Low	Time Window	295	269	<0.001 ***	>10^4^
Posterior	Low	Task	295	0.66	0.509	0.025
Posterior	Low	Timewindow × Task	295	0.31	0.406	0.006

**Table 3:** A summary of all pairwise comparisons for the two electrode clusters. An FDR correction was applied across comparisons. Significance after the FDR correction is denoted in the corresponding column of the table. When marking significance.

Pairwise Comparisons for Electrode Cluster										
Cluster	PLV		Time Window	Frequency Band	Task	df	t	*p*	FDR	Cohen’s d
	Mean	SD								
Frontal	0.105	0.005	Pre-Stimulus	High	Active WM+	59	−0.61	0.54	n.s.	−0.08
Posterior	0.109	0.004								
Frontal	0.106	0.005	Pre-Stimulus	High	Active WM-	59	−0.35	0.731	n.s.	−0.05
Posterior	0.109	0.003								
Frontal	0.106	0.005	Pre-Stimulus	High	Passive WM-	59	−1.19	0.24	n.s.	−0.15
Posterior	0.109	0.003								
Frontal	0.114	0.002	Pre-Stimulus	Low	Active WM+	59	1.11	0.271	n.s.	0.14
Posterior	0.116	0.003								
Frontal	0.114	0.002	Pre-Stimulus	Low	Active WM-	59	−0.74	0.464	n.s.	−0.10
Posterior	0.114	0.002								
Frontal	0.114	0.003	Pre-Stimulus	Low	Passive WM-	59	1.64	0.107	n.s.	0.21
Posterior	0.115	0.002								
Frontal	0.176	0.005	Post-Stimulus	High	Active WM+	59	−1.4	0.168	n.s.	−0.18
Posterior	0.179	0.004								
Frontal	0.177	0.006	Post-Stimulus	High	Active WM-	59	−1.51	0.134	n.s.	−0.19
Posterior	0.180	0.004								
Frontal	0.177	0.005	Post-Stimulus	High	Passive WM-	59	−0.86	0.578	n.s.	−0.11
Posterior	0.179	0.004								
Frontal	0.186	0.006	Post-Stimulus	Low	Active WM+	59	0.66	0.51	n.s.	0.09
Posterior	0.187	0.003								
Frontal	0.184	0.005	Post-Stimulus	Low	Active WM-	59	−0.45	0.658	n.s.	−0.06
Posterior	0.186	0.003								
Frontal	0.185	0.006	Post-Stimulus	Low	Passive WM-	59	−1.91	0.06	n.s.	−0.25
Posterior	0.187	0.003								

**Table 4:** A summary of all pairwise comparisons for the two frequency bands. An FDR correction was applied across comparisons. Significance after the FDR correction is denoted in the corresponding column of the table. When marking significance, * denotes significance after FDR correction.

Pairwise Comparisons for Frequency Band.										
Frequency	PLV		Time window	Cluster	Task	df	t	*p*	FDR	Cohen’s d
	Mean	SD								
High	0.105	0.005	Pre-Stimulus	Frontal	Active WM+	59	−9.8	<0.001	*	−1.27
Low	0.114	0.002								
High	0.106	0.005	Pre-Stimulus	Frontal	Active WM-	59	−10.2	<0.001	*	−1.32
Low	0.114	0.002								
High	0.106	0.005	Pre-Stimulus	Frontal	Passive WM-	59	−12.2	<0.001	*	−1.58
Low	0.114	0.003								
High	0.109	0.004	Pre-Stimulus	Posterior	Active WM+	59	−10.5	<0.001	*	−1.36
Low	0.116	0.003								
High	0.109	0.003	Pre-Stimulus	Posterior	Active WM-	59	−12.4	<0.001	*	−1.60
Low	0.114	0.002								
High	0.109	0.003	Pre-Stimulus	Posterior	Passive WM-	59	−10.4	<0.001	*	−1.34
Low	0.115	0.002								
High	0.176	0.005	Post-Stimulus	Frontal	Active WM+	59	−6.9	<0.001	*	−0.89
Low	0.186	0.006								
High	0.177	0.006	Post-Stimulus	Frontal	Active WM-	59	−8.8	<0.001	*	−1.14
Low	0.184	0.005								
High	0.177	0.005	Post-Stimulus	Frontal	Passive WM-	59	−4.1	<0.001	*	−0.53
Low	0.185	0.006								
High	0.179	0.004	Post-Stimulus	Posterior	Active WM+	59	−10.4	<0.001	*	−1.34
Low	0.187	0.003								
High	0.180	0.004	Post-Stimulus	Posterior	Active WM-	59	−12.9	<0.001	*	−1.67
Low	0.186	0.003								
High	0.179	0.004	Post-Stimulus	Posterior	Passive WM-	59	−12.9	<0.001	*	−1.67
Low	0.187	0.003								

## Data Availability

The data presented in this study are available on request from the corresponding author. The data are not publicly available due to privacy restrictions.

## Supplementary Materials

The following supporting information can be downloaded at: https://www.mdpi.com/article/10.3390/brainsci12091131/s1, Figure S1: Mean gamma amplitude (across the low gamma band) by theta phase plotted separately for each electrode in our two clusters. Note that electrodes in the frontal cluster show mostly the same response to theta phase, while electrodes in the posterior cluster differ in their specific tuning. The scalp maps on the top left of each plot indicate which electrode corresponds to each line in the plot. Table S1: A summary of the replication sample results from the task x time-window ANOVAS. When marking significance, *** denotes *p* < 0.001. Table S2: The resul from the replication sample for the linear mixed effects models using the focused contrast testing whether PLV in the Active WM+ condition is higher than the other two conditions. When marking significance, denotes *p* < 0.001. Table S3: A summary of the replication sample results from pairwise comparisons of the mean PLV in the frontal and posterior clusters. A false discovery rate correction was to correct for multiple comparisons. The FDR column denotes which comparisons were significant after correction. When marking significance, * significance after FDR correction. Table S4: A summary of the replication sample results from pairwise comparisons of the mean PLV across the high and low frequency bands. A false discovery rate correction was to correct for multiple comparisons. The FDR column denotes which comparisons were significant after correction. When marking significance, * significance after FDR correction.
