# Supplementary material for: Is Cortical Theta-Gamma Phase-Amplitude Coupling Memory-Specific?
Source: Brain Sci. 2022 Aug 25;12(9):1131. doi: 10.3390/brainsci12091131 (PMC9496728; doi:10.3390/brainsci12091131)
Supplement: Supplementary file 1 [file brainsci-12-01131-s001.zip › brainsci-1850933-SI.pdf]

Is Cortical Theta-Gamma Phase-Amplitude Coupling Memory-Specific?

Orestis Papaioannou [1,*] , Laura P. Crespo [1], Kailey Clark [1], Nicole N. Ogbuagu [1], Luz Maria Alliende [1] , Steven M. Silverstein [2] and Molly A. Erickson [1]

[1] Department of Psychiatry & Behavioral Neuroscience, University of Chicago, Chicago, IL 60637, USA

[2] Departments of Psychiatry, Neuroscience and Ophthalmology, University of Rochester, Rochester, NY 14627, USA

***** Correspondence: opapaioannou@uchicago.edu

Section S1

Differences in the consistency of theta-gamma amplitude across electrode cluster

One interesting and unexpected finding is that the consistency of coupling across neighboring channels differed as a function of electrode cluster. This pattern was most evident in the low gamma band (see figure AI1), but in general frontal electrodes showed consistent coupling, with gamma amplitude from most frontal electrodes peaking at the same theta phase. Conversely, electrodes in the posterior cluster show much more variability in the particular theta phase where gamma amplitude is maximal. It seems that theta-gamma phase-amplitude coupling shifts in phase-space (although crucially remains comparable in size) as you move from posterior to occipital electrodes. Furthermore, posterior, but not occipital electrodes show some lateralization effects, with gamma amplitude for electrodes on different hemispheres peaking at slightly different phases.

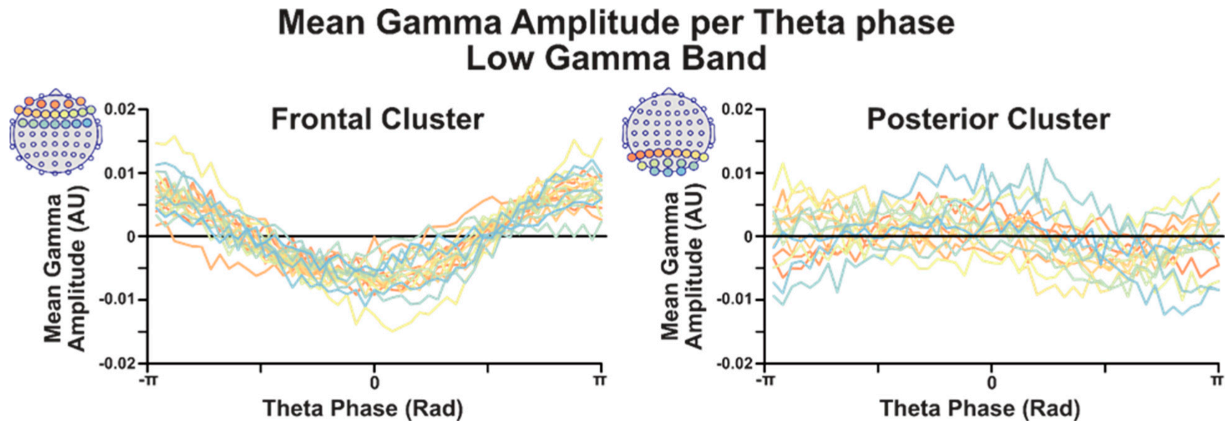

Figure S1: Mean gamma amplitude (across the low gamma band) by theta phase plotted separately for each electrode in our two clusters. Note that electrodes in the frontal cluster show mostly the same response to theta phase, while electrodes in the posterior cluster differ in their specific tuning. The scalp maps on the top left of each plot indicate which electrode corresponds to each line in the plot.

It is unclear if there is any functional significance to this difference, as both clusters show similar overall levels of coupling. It is possible that this indicates different levels of coordination across frontal and posterior areas of the brain. For example, it could be that the more localized coupling seen in posterior electrodes may result from the more localized stimulus representations found in posterior visual areas, with different stimulus representations peaking at different theta phases to keep visual objects separable. Conversely, coupling in frontal electrodes may represent activity in the prefrontal cortex. Thus, theta signaling here may not act in a more distributed manner to gate higher order processes such as attentional control across larger swathes of the prefrontal cortex. Note however that this interpretation is purely hypothetical, and we can not make any strong claims as to the source or significance of these effects.

Section S2

Replication Sample Results

Given the exploratory nature of our analyses, we also collected data from a secondary sample of 20 participants to act as a replication sample for our main findings. All signal processing and statistical analyses were performed in identical manner to the original primary sample. The table below summarizes the results of main statistical comparisons for this replication sample.

*Table S1: A summary of the replication sample results from the task x time-window ANOVAS. When marking significance, *** denotes p<0.001.*

Task by Time-Window ANOVAs

Cluster	Frequency Band	Effect	dfs	F	p(GG)	η^2	BF10
Frontal	High	Time Window	1, 19	13396	<0.001***	0.980	$>10^{116}$
Frontal	High	Task	183, 34.8	1.07	0.35	0.008	0.079
Frontal	High	Timewindow X Task	1.74, 33.1	0.03	0.955	0.000	--
Frontal	Low	Time Window	1, 19	5634	<0.001***	0.986	$>10^{115}$
Frontal	Low	Task	1.67, 31.8	0.63	0.508	0.006	0.079
Frontal	Low	Timewindow X Task	1.58, 30.0	0.385	0.634	0.002	--
Posterior	High	Time Window	1, 19	32234	<0.001***	0.990	$>10^{128}$
Posterior	High	Task	1.81,34.3	0.09	0.893	0.001	0.081
Posterior	High	Timewindow X Task	184, 34.9	0.47	0.61	0.004	--
Posterior	Low	Time Window	1, 19	28648	<0.001***	0.995	$>10^{135}$
Posterior	Low	Task	1.91,112.7	2.62	0.09	0.044	0.079
Posterior	Low	Timewindow X Task	1.91,112.6	0.27	0.741	0.003	--

Table S2: The resul from the replication sample for the linear mixed effects models using the focused contrast testing whether PLV in the Active WM+ condition is higher than the other two conditions. When marking significance, denotes p<0.001.

**Contrast Analysis Results**						
**Cluster**	**Frequency Band**	**Effect**	**df**	**t**	**p**	**Cohen's d**
Frontal	High	Time Window	95	127	<0.001***	$>10^4$
Frontal	High	Task	95	-1.10	0.271	0.124
Frontal	High	Timewindow X Task	95	-0.05	0.961	0.000
Frontal	Low	Time Window	95	114	<0.001***	$>10^4$
Frontal	Low	Task	95	0.27	0.782	0.007
Frontal	Low	Timewindow X Task	95	0.6	0.550	0.037
Posterior	High	Time Window	95	149	<0.001***	$>10^4$
Posterior	High	Task	95	0.51	0.605	0.027
Posterior	High	Timewindow X Task	95	-0.5	0.617	0.026
Posterior	Low	Time Window	95	164	<0.001***	>10^4
Posterior	Low	Task	95	1.57	0.119	0.253
Posterior	Low	Timewindow X Task	95	-0.33	0.742	0.011

*Table S3: A summary of the replication sample results from pairwise comparisons of the mean PLV in the frontal and posterior clusters. A false discovery rate correction was to correct for multiple comparisons. The FDR column denotes which comparisons were significant after correction. When marking significance, * significance after FDR correction.*

Pairwise Comparisons Between Electrode Clusters

Cluster	PLV		Time window	Frequency Band	Task	df	t	p	FDR	Cohen's d
	Mean	SD								
Frontal	0.105	0.005	Pre-Stimulus	High	Active WM+	59	−0.70	0.49	n.s.	−0.09
Posterior	0.109	0.004								
Frontal	0.106	0.005	Pre-Stimulus	High	Active WM-	59	2.05	0.05	n.s.	0.26
Posterior	0.109	0.003								
Frontal	0.106	0.005	Pre-Stimulus	High	Passive WM-	59	−0.19	0.85	n.s.	−0.02
Posterior	0.109	0.003								
Frontal	0.114	0.002	Pre-Stimulus	Low	Active WM+	59	0.36	0.72	n.s.	0.05
Posterior	0.116	0.003								
Frontal	0.114	0.002	Pre-Stimulus	Low	Active WM-	59	−1.01	0.29	n.s.	−0.13
Posterior	0.114	0.002								
Frontal	0.114	0.003	Pre-Stimulus	Low	Passive WM-	59	−2.64	0.02	n.s.	−0.34
Posterior	0.115	0.002								
Frontal	0.176	0.004	Post-Stimulus	High	Active WM+	59	−2.02	0.06	n.s.	−0.26
Posterior	0.179	0.006								
Frontal	0.177	0.004	Post-Stimulus	High	Active WM-	59	1.02	0.32	n.s.	0.13
Posterior	0.180	0.005								
Frontal	0.177	0.004	Post-Stimulus	High	Passive WM-	59	-0.91	0.38	n.s.	−0.12
Posterior	0.179	0.006								
Frontal	0.186	0.003	Post-Stimulus	Low	Active WM+	59	0.90	0.38	n.s.	0.12
Posterior	0.187	0.005								
Frontal	0.184	0.003	Post-Stimulus	Low	Active WM-	59	-1.76	0.09	n.s.	−0.23
Posterior	0.186	0.006								
Frontal	0.185	0.003	Post-Stimulus	Low	Passive WM-	59	-1.24	0.23	n.s.	−0.16
Posterior	0.187	0.005								

*Table S4: A summary of the replication sample results from pairwise comparisons of the mean PLV across the high and low frequency bands. A false discovery rate correction was to correct for multiple comparisons. The FDR column denotes which comparisons were significant after correction. When marking significance, * significance after FDR correction.*

Pairwise Comparisons Between Frequency Band

Frequency	PLV		Time window	Cluster	Task	df	t	p	FDR	Cohen's d
	Mean	SD								
Low	0.114	0.005	Pre-Stimulus	Frontal	Active WM+	59	−5.87	<0.001	*	−0.76
High	0.105	0.002								
Low	0.114	0.005	Pre-Stimulus	Frontal	Active WM-	59	−5.84	<0.001	*	−0.75
High	0.106	0.002								
Low	0.114	0.005	Pre-Stimulus	Frontal	Passive WM-	59	−8.74	<0.001	*	−1.13
High	0.106	0.003								
Low	0.116	0.004	Pre-Stimulus	Posterior	Active WM+	59	−5.38	<0.001	*	−0.69
High	0.109	0.003								
Low	0.114	0.003	Pre-Stimulus	Posterior	Active WM-	59	−6.27	<0.001	*	−0.81
High	0.109	0.002								
Low	0.115	0.003	Pre-Stimulus	Posterior	Passive WM-	59	−8.44	<0.001	*	−1.09
High	0.109	0.002								
Low	0.186	0.005	Post-Stimulus	Frontal	Active WM+	59	−2.97	<0.001	*	−0.38
High	0.176	0.006								
Low	0.184	0.006	Post-Stimulus	Frontal	Active WM-	59	−4.92	<0.001	*	−0.64
High	0.177	0.005								
Low	0.185	0.005	Post-Stimulus	Frontal	Passive WM-	59	−6.6	<0.001	*	−0.85
High	0.177	0.006								
Low	0.187	0.004	Post-Stimulus	Posterior	Active WM+	59	−6.57	<0.001	*	−0.85
High	0.179	0.003								
Low	0.186	0.004	Post-Stimulus	Posterior	Active WM-	59	−7.03	<0.001	*	−0.91
High	0.180	0.003								
Low	0.187	0.004	Post-Stimulus	Posterior	Passive WM-	59	−8.06	<0.001	*	−1.04
High	0.179	0.003								
